# Supplementary material for: Mapping global inputs and impacts from of human sewage in coastal ecosystems
Source: PLoS One. 2021 Nov 10;16(11):e0258898. doi: 10.1371/journal.pone.0258898 (PMC8580218; doi:10.1371/journal.pone.0258898)
Supplement: S1 File — (PDF) [file pone.0258898.s001.pdf]

S1 File

Supporting Information for: Mapping global inputs and impacts from of human sewage in coastal ecosystems

S1 Text

Throughout our process, we compared intermediate results to previously-reported literature to ensure that our approach is within probable bounds. We performed two main types of comparisons. The first step is to corroborate that our national-level sanitation factors accurately represent the spatial distribution of these values. For that, we compared spatially explicit and anonymized measurements reported by USAID Domestic Household Surveys (DHS) to our WHO-derived estimates. These results are shown in Fig. S5.

We then benchmarked our pourpoint-level effluent against in situ measurements of annual N effluent in 83 rivers across the world from the Global Nutrient Export from Watersheds 2 (NEWS2) [1]. The NEWS2 model uses in situ measurements to calibrate their model; these data report position (longitude, latitude) and total N effluent (Kg N yr^{-1}). Our pourpoint-level analysis required us to assign more than one pourpoint to a given river mouth. Therefore, we used different buffer sizes around the reported river mouths (0.1, 0.25, 0.5, and 1 degree), and attributed all pourpoints within this buffer to the river. We then summed total N from all pourpoints to obtain river-level estimates (Fig. S6).

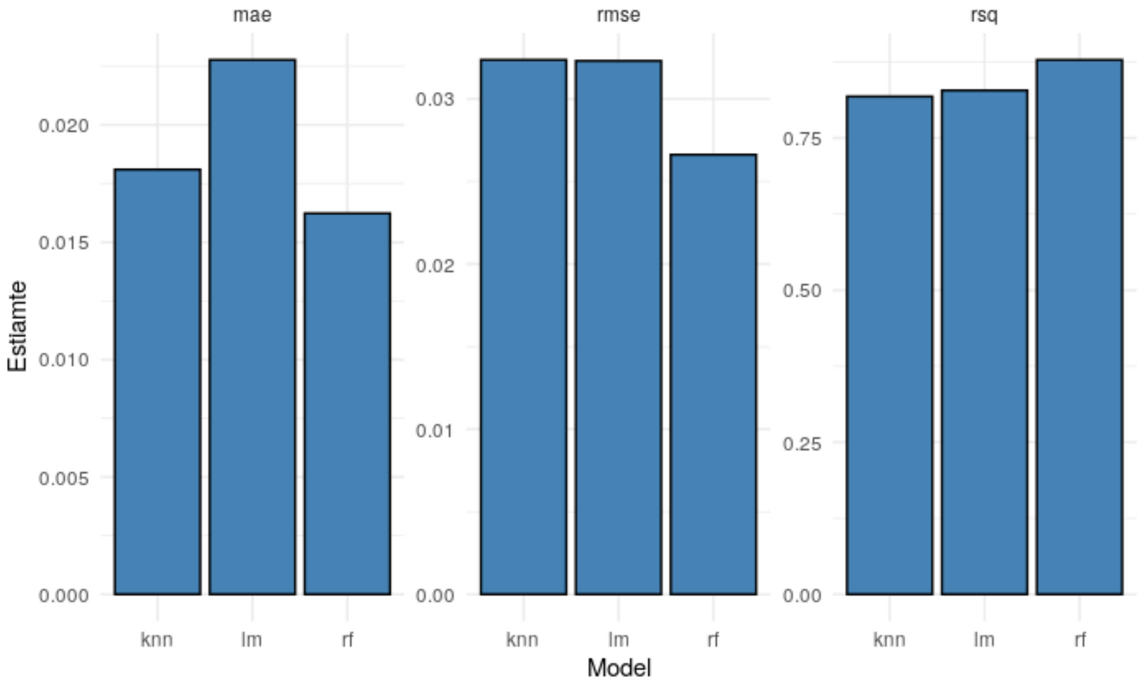

S1 Fig. Model performance based on a 70/30 data split for training and testing. The x-axis shows three model types (K-nearest neighbors, linear model, and random forest), with the y-axis showing the out-of-sample estimate of different performance indicators. Panels show the Mean Absolute Error (mae), the Root-Mean-Squared Error (rmse), and the R squared (rsq). The random forest has the lowest out-of-sample errors, and the highest out-of-sample R-squared.

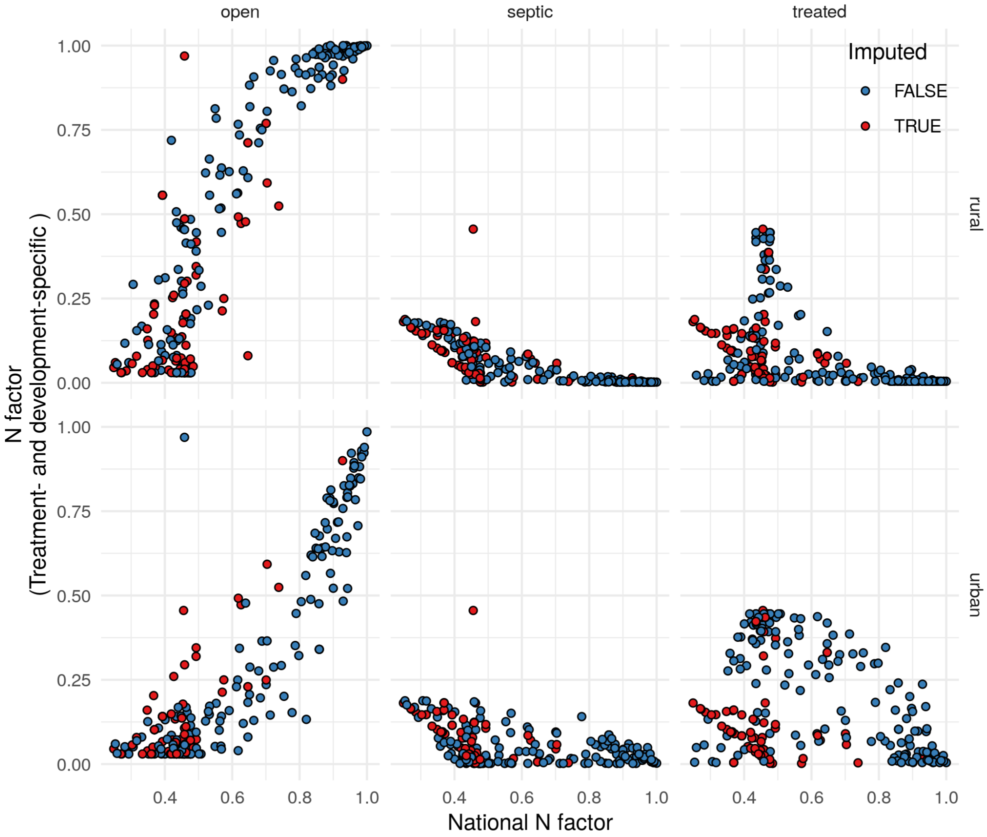

S2 Fig. Comparison of rural- and urban-level nitrogen factors for each country and treatment time versus aggregate national-level values. The WHO-UNICEF data contain information that aggregates access to sanitation types at the national level, and for some countries it distinguishes between rural and urban areas.

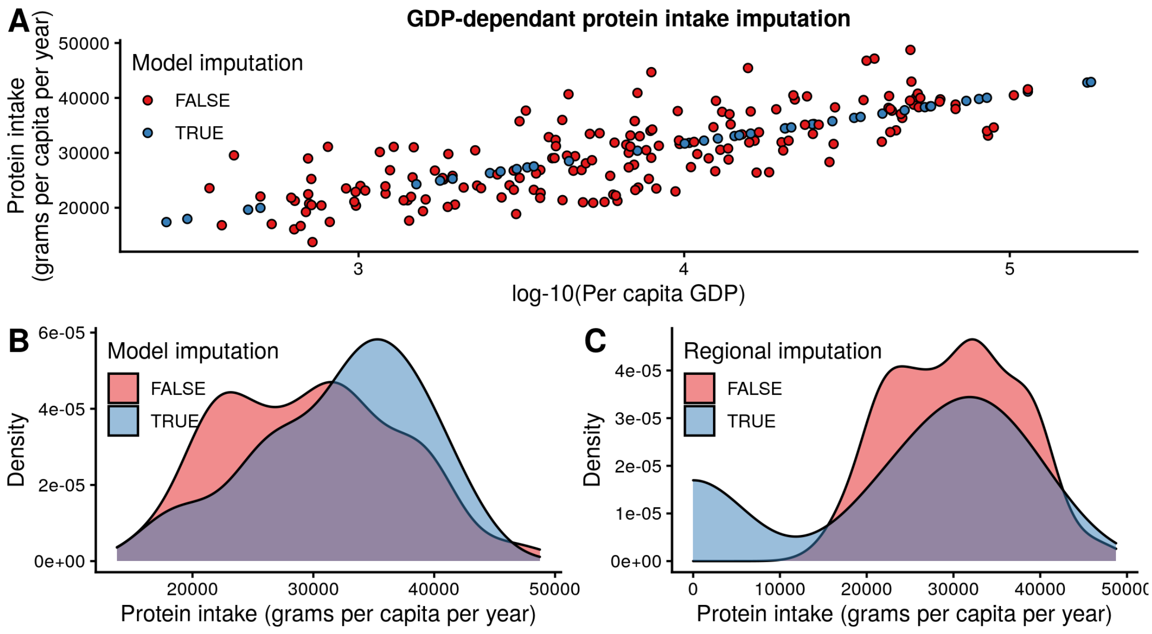

S3 Fig. Imputation of protein intake as a function of per capita GDP. A) The relationship between log-transformed per capita GDP and protein intake for each country for which data are known (red; not modeled and thus model imputation is false) and imputed (blue; modeled and thus true). Density distribution plots for known and imputed protein intake values at the B) country or C) geopolitical region means.

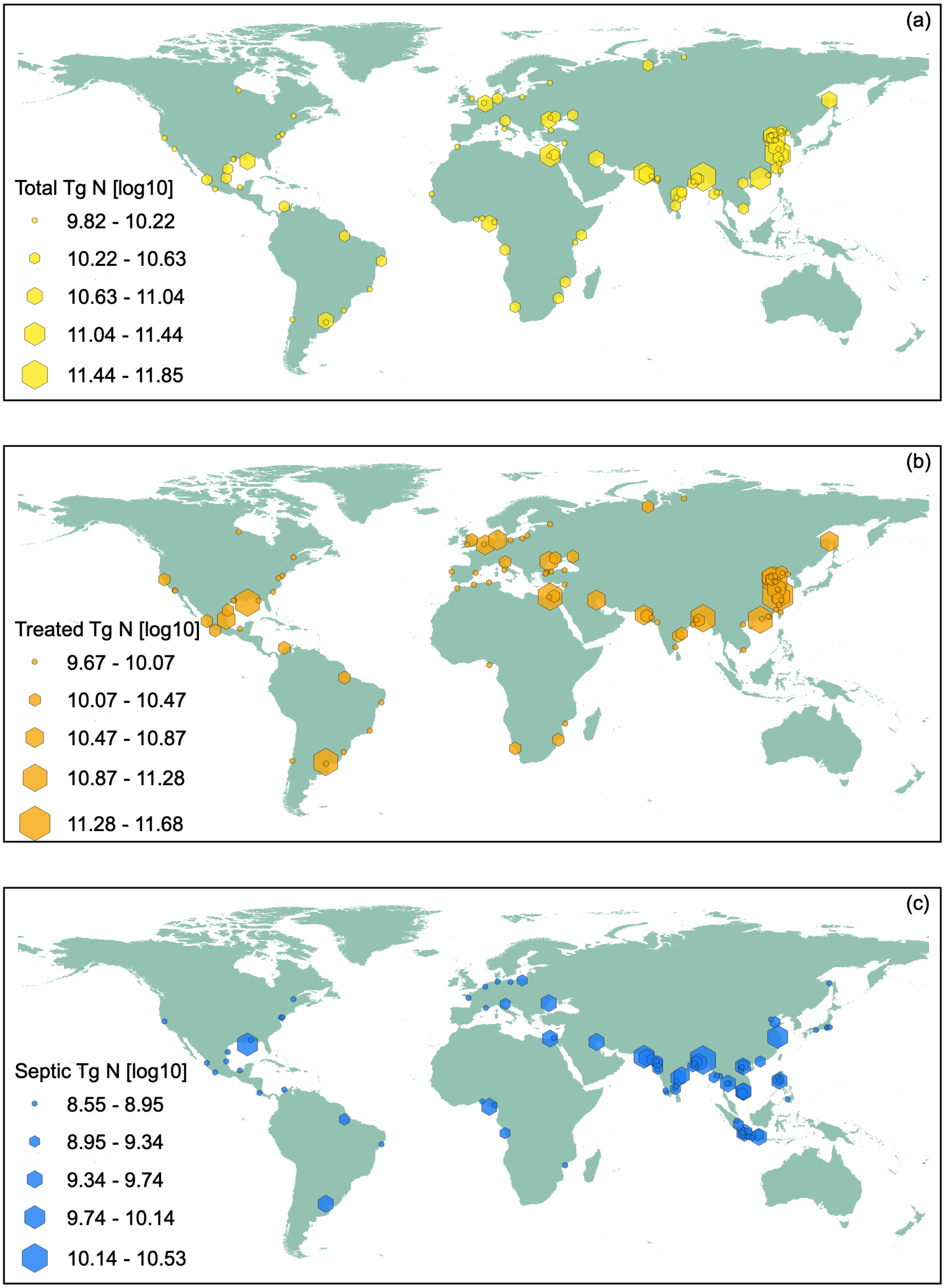
(a)
Total Tg N [log10]
9.82 - 10.22
10.22 - 10.63
10.63 - 11.04
11.04 - 11.44
11.44 - 11.85
(b)
Treated Tg N [log10]
9.67 - 10.07
10.07 - 10.47
10.47 - 10.87
10.87 - 11.28
11.28 - 11.68
(c)
Septic Tg N [log10]
8.55 - 8.95
8.95 - 9.34
9.34 - 9.74
9.74 - 10.14
10.14 - 10.53

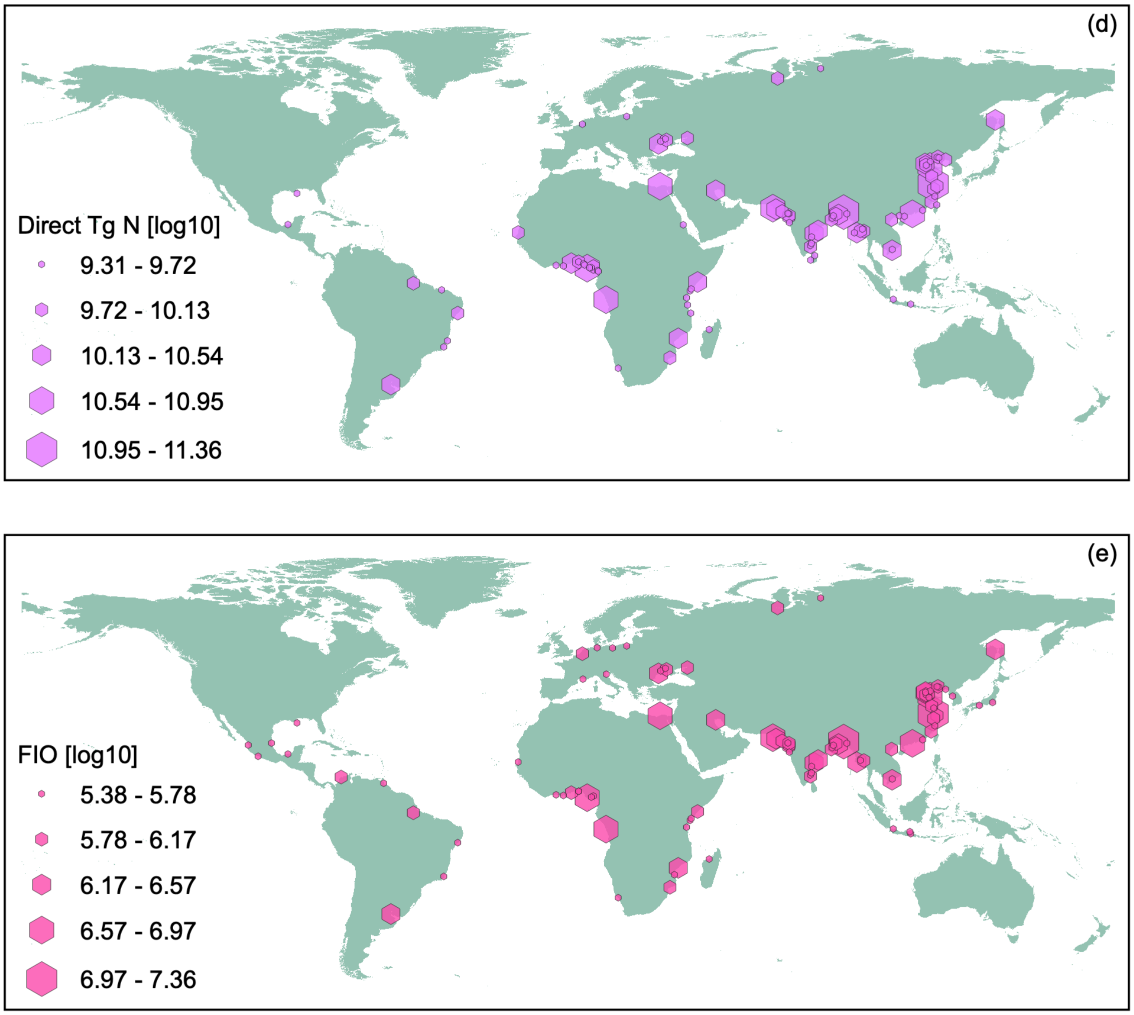

S4 Fig. Coastal pourpoint locations of the top 100 watersheds for wastewater inputs globally for (a) total N (yellow), (b) treated (orange), (c) septic (blue), (d) direct (purple), and (e) and FIO inputs globally (pink). Note that FIO estimates are unitless.

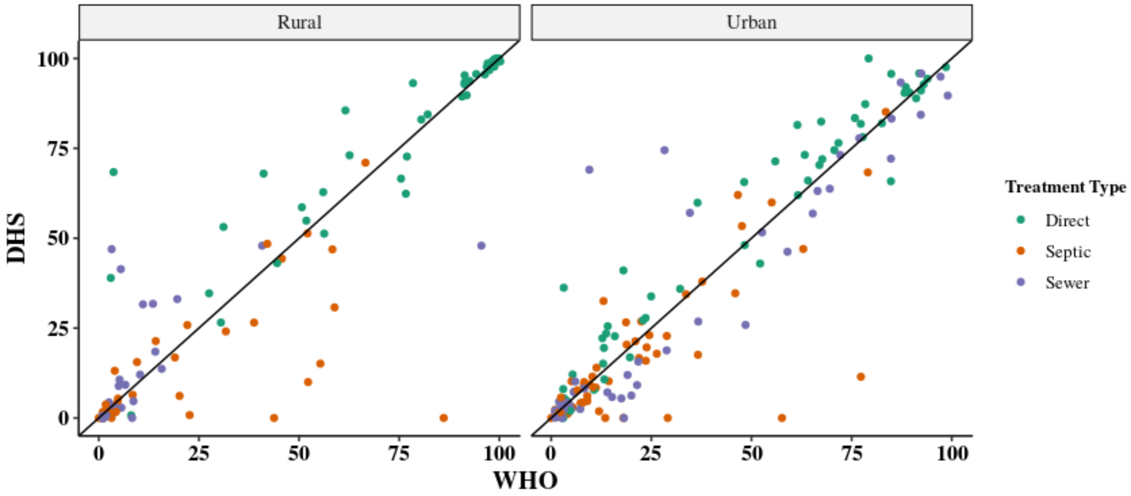

S5 Fig. Comparison of wastewater treatment types (direct, septic, sewer) between the WHO and DHS survey datasets for rural and urban settings.

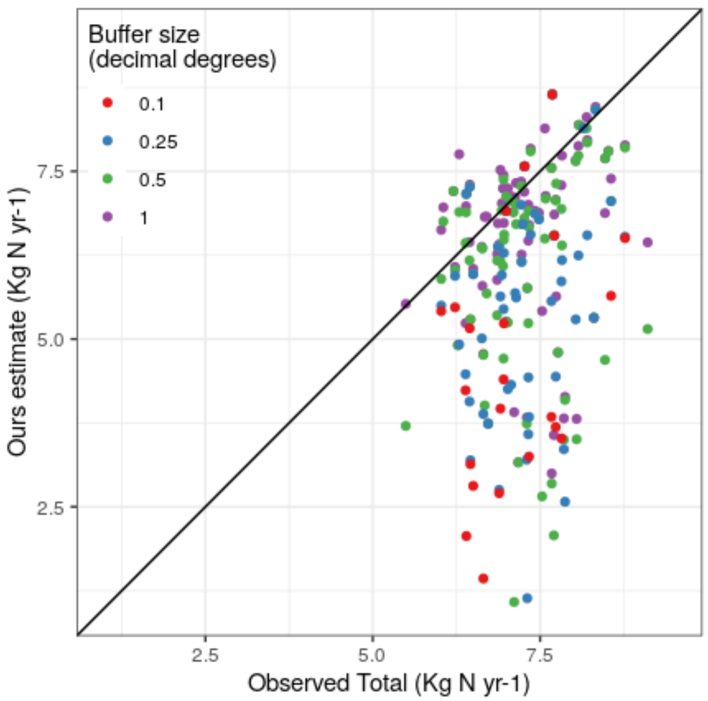

S6 Fig. Comparison of observed N and estimated N for 83 rivers in the world for four buffer sizes (colors). Each point represents one river and buffer size, and the dark line indicates a 1:1 correspondence. Values above the line represent modeled values that are greater than *in situ* measured values. Different buffer sizes have little effect on the estimated N.

Treatment category	JMP listing
Direct	Unimproved<br>Open defecation<br>Including shared latrines and other
Septic	Including shared septic tanks
Treated	Including shared sewer connections

S1 Table Treatment facility categories used in this paper and the corresponding categories from the original JMP data. For a given pixel, the units of these categories are the proportion of the rural or urban population using that facility. When added together they sum to 100%.

Independent Variable	Dependent Variable<br>Per capita protein consumption (grams / person / year)
log10 (Per capita GDP)	8,983.259*** (584.407)
Constant	-4,261.140* (2,245.397)
Observations	174
R^2	0.579
Residual Std. Error	4,760.402 (df = 172)
F Statistic	236.285*** (df = 1; 172)
Note:	*p<0.1; **p<0.05; ***p<0.01

S2 Table Coefficient estimates for protein consumption as a function of per capita gdp (log-10 transformed). These coefficients were used to impute protein consumption for 37 countries, shown in Extended Data Fig. 3.

ISO3	Region	Source of Imputation	Protein consumption (grams per person per year)
ABW	Caribbean	GDP model	35250.61
AIA	Caribbean	Geopolitical regional median	30426.4
ALA	Northern Europe	Geopolitical regional median	39949.25
AND	Southern Europe	GDP model	37141.02
ASM	Polynesia	GDP model	32218.99
ATA	NA	Geopolitical regional median	0
ATF	Eastern Africa	Geopolitical regional median	20916.33
BDI	Eastern Africa	GDP model	17387.55
BES	Caribbean	Geopolitical regional median	30426.4
BHR	Western Asia	GDP model	35206.6
BLM	Caribbean	Geopolitical regional median	30426.4
BTN	Southern Asia	GDP model	26313.08
BVT	NA	Geopolitical regional median	0
CCK	South-Eastern Asia	Geopolitical regional median	24557.2
COD	Middle Africa	GDP model	19641.8
COK	Polynesia	Geopolitical regional median	33284.35
COM	Eastern Africa	GDP model	24283.29
CPT	NA	Geopolitical regional median	0
CUW	Caribbean	GDP model	34465.36
CXR	South-Eastern Asia	Geopolitical regional median	24557.2
CYM	Caribbean	GDP model	39454.83
ERI	Eastern Africa	Geopolitical regional median	20916.33
ESH	Northern Africa	Geopolitical regional median	35744.45
ETH	Eastern Africa	GDP model	19980.75
FLK	South America	Geopolitical regional median	26997.22
FRO	Northern Europe	GDP model	38312.37

GGY	Northern Europe	Geopolitical regional median	39949.25
GIB	Southern Europe	Geopolitical regional median	38943.68
GLP	Caribbean	Geopolitical regional median	30426.4
GNQ	Middle Africa	GDP model	34450.5
GRL	Northern America	GDP model	37753.73
GUF	South America	Geopolitical regional median	26997.22
GUM	Micronesia	GDP model	36361.09
HMD	NA	Geopolitical regional median	0
IMN	Northern Europe	GDP model	39805.58
IOT	Southern Asia	Geopolitical regional median	22947.55
JEY	Northern Europe	Geopolitical regional median	39949.25
LBY	Northern Africa	GDP model	31811.31
LIE	Western Europe	GDP model	42794.9
LUX	Western Europe	GDP model	41153.5
MAF	Caribbean	Geopolitical regional median	30426.4
MCO	Western Europe	GDP model	42895.82
MHL	Micronesia	GDP model	27342.68
MNE	Southern Europe	GDP model	30382.92
MNP	Micronesia	GDP model	33060.29
MSR	Caribbean	Geopolitical regional median	30426.4
MTQ	Caribbean	Geopolitical regional median	30426.4
MYT	Eastern Africa	Geopolitical regional median	20916.33
NFK	Australia and New Zealand	Geopolitical regional median	37981.9
NIU	Polynesia	Geopolitical regional median	33284.35
NRU	Micronesia	GDP model	31687.19
PCN	Polynesia	Geopolitical regional median	33284.35
PLW	Micronesia	GDP model	32600.77
PNG	Melanesia	GDP model	26606.65
PRI	Caribbean	GDP model	35759.69

PSE	Western Asia	GDP model	27052.4
QAT	Western Asia	GDP model	40023.45
REU	Eastern Africa	Geopolitical regional median	20916.33
RUS	Eastern Europe	GDP model	33507.29
SDN	Northern Africa	GDP model	25281.9
SGP	South-Eastern Asia	GDP model	38459.89
SGS	NA	Geopolitical regional median	0
SHN	Western Africa	Geopolitical regional median	22869.08
SJM	Northern Europe	Geopolitical regional median	39949.25
SMR	Southern Europe	GDP model	38482.78
SOM	Eastern Africa	GDP model	17964.8
SPM	Northern America	Geopolitical regional median	38306.75
SSD	Northern Africa	GDP model	24937.06
SXM	Caribbean	Geopolitical regional median	30426.4
SYC	Eastern Africa	GDP model	33192.34
SYR	Western Asia	Geopolitical regional median	32537.92
TCA	Caribbean	GDP model	34627.12
TKL	Polynesia	Geopolitical regional median	33284.35
TON	Polynesia	GDP model	28494.19
TUV	Polynesia	GDP model	27525.3
UMI	NA	Geopolitical regional median	0
VAT	Southern Europe	Geopolitical regional median	38943.68
VGB	Caribbean	Geopolitical regional median	30426.4
VIR	Caribbean	GDP model	36539.19
WLF	Polynesia	Geopolitical regional median	33284.35
XAD	NA	Geopolitical regional median	0
XCA	NA	Geopolitical regional median	0
XCL	NA	Geopolitical regional median	0

XKO	NA	Geopolitical regional median	0
XNC	NA	Geopolitical regional median	0
XPI	NA	Geopolitical regional median	0
XSP	NA	Geopolitical regional median	0

S3 Table Jurisdictions for which protein consumption values were imputed, with source of imputation and resulting values.

basin_id	Country	River or City	Total N (g)	N (g/m2)	Total N Rank	Total N(g/m^2) Rank
**Top 25 Watersheds Ranked by Total N (g)**						
al_12731	China	Yangtze River	7.1E+11	0.37088357	1	8384
al_13237	Bangladesh	Meghna River	3.62E+11	0.22758124	2	11432
al_13478	China	Shenwan	1.89E+11	0.41766363	3	7755
af_16862	Egypt	Nile River	1.59E+11	0.05261516	4	23026
al_11370	China	Binzhou	1.26E+11	0.34718395	5	8766
al_11956	Pakistan	Keti Bandar	1.19E+11	0.15777384	6	14002
na_75069	USA	Mississippi River	1.01E+11	0.03152891	7	27602
sa_06694	Argentina	Rio Parana	9.6E+10	0.03640112	8	26329
al_09132	Russia	Amur River	9.04E+10	0.04466534	9	24424
af_15969	Niger	Niger River	8.6E+10	0.03948781	10	25580
eu_30333	Romania	Danube River	8.45E+10	0.10625456	11	17059
al_10872	China	Tianjin	7.66E+10	0.73071692	12	5083
eu_27295	Netherlands	Rhine River	7.56E+10	0.39064582	13	8098
al_10213	China	Dongli District	6.1E+10	0.69071214	14	5320
al_11575	China	Shanghai	5.86E+10	2.50511331	15	1564
af_09315	Iraq	Shatt al-Arab	5.37E+10	0.06126411	16	21762
al_11201	China	Chenjiagang	5.35E+10	0.88730868	17	4295
al_14328	India	Krishna River	4.81E+10	0.18849689	18	12668
al_12164	India	Dayapar	4.71E+10	0.11670111	19	16314
al_14070	India	Godavari River	4.16E+10	0.13384341	20	15259
af_17918	DRC	Congo River	4.14E+10	0.01115765	21	36679
al_09282	China	Zhao Quan He	3.75E+10	0.18380861	22	12861
al_14362	Myanmar	Pathein	3.64E+10	0.09281258	23	18184
eu_27181	Ukraine	Dnieper River	3.49E+10	0.07073249	24	20529
al_11174	China	Huanghua	3.47E+10	0.62451202	25	5787
**Top 25 Watersheds Ranker by N per Area (g/m^2)**						
ai_07771	China	Taozi Bay	86730972	142.5221851	3715	1

al_12918	China	Hong Kong	1.54E+09	119.9969052	374	2
al_12913	China	Tsing Yi	2.03E+09	85.4788765	286	3
al_12915	Hong Kong	Hong Kong	2.39E+09	81.39985878	257	4
eu_30871	Turkey	Zeytinburnu/İstanbul	2.82E+09	68.83582257	218	5
ai_09953	China	Qiantang River	40233778	66.00517693	6094	6
al_10759	China	Qingdao	1.05E+09	61.45810926	550	7
eu_30851	Turkey	Istanbul	7.57E+08	61.24758241	703	8
na_70998	USA	New York	1.97E+09	56.73549546	302	9
al_12503	China	Xiamen Port	2.82E+09	56.33175679	220	10
ai_07801	China	Kongtong Island / Zhifu Bay	34158623	56.13089252	6756	11
al_15910	Singapore	Singapore	6.97E+08	54.30275212	749	12
na_71048	USA	Brooklyn	7.66E+08	52.46965126	695	13
na_71080	USA	New York	7.32E+08	52.30801973	720	14
al_12911	Hong Kong	Hong Kong	30487394	49.94564304	7203	15
na_70978	USA	New York	1.51E+09	47.01151487	380	16
al_15872	Singapore	Singapore	6.74E+08	45.94213325	771	17
al_10768	China	Jiaozhou Wan/Qingdao	1.28E+09	45.6801241	450	18
eu_29729	Spain	Barcelona	6.96E+08	45.4419491	750	19
al_15899	Singapore	Singapore	1.43E+09	44.21169406	406	20
al_15909	Singapore	Singapore	8.56E+08	41.15963562	638	21
eu_30892	Turkey	Istanbul	4.82E+09	41.12931255	145	22
af_03174	Turkey	Istanbul	5.87E+08	38.28465088	860	23
af_18168	Mozambique	Island of Mozambique	32539763	37.98566968	6942	24
al_12903	Hong Kong	Hong Kong	6.02E+08	37.90202359	845	25

S4 Table Top 25 watersheds or coastal urban locations of both N input by total amount (rank) and N input normalized by watershed size (normalized rank).

ISO3	Country Name	Direct	Septic	Sewer	Total
ABW	Aruba	5.65E+05	2.27E+06	6.05E+07	6.33E+07
AFG	Afghanistan	8.71E+09	5.55E+08	7.11E+08	9.97E+09
AGO	Angola	3.18E+09	1.06E+09	3.22E+09	7.47E+09
AIA	Anguilla	7.77E+05	2.44E+06	3.01E+06	6.22E+06
ALA	Åland Islands	2.62E+06	1.35E+05	3.85E+07	4.12E+07
ALB	Albania	1.56E+08	2.10E+07	4.73E+09	4.91E+09
AND	Andorra	5.71E+06	1.08E+07	1.07E+08	1.23E+08
ARE	United Arab Emirates	4.72E+08	1.17E+08	3.98E+08	9.87E+08
ARG	Argentina	5.70E+09	1.68E+09	3.28E+10	4.02E+10
ASM	American Samoa	3.47E+06	2.03E+06	1.17E+07	1.72E+07
ATG	Antigua & Barbuda	4.87E+06	3.80E+06	3.83E+07	4.69E+07
AUS	Australia	6.83E+08	5.22E+08	1.88E+09	3.08E+09
AUT	Austria	5.29E+08	2.41E+08	1.25E+10	1.33E+10
BDI	Burundi	2.95E+09	1.53E+08	3.50E+08	3.46E+09
BEL	Belgium	3.90E+08	1.29E+08	5.20E+08	1.04E+09
BEN	Benin	4.70E+09	6.42E+07	2.24E+08	4.99E+09
BES	Caribbean Netherlands	5.23E+06	1.01E+06	4.29E+06	1.05E+07
BFA	Burkina Faso	1.28E+10	1.11E+08	4.95E+08	1.34E+10
BGD	Bangladesh	8.38E+10	5.24E+09	1.71E+10	1.06E+11
BGR	Bulgaria	3.24E+09	4.12E+08	5.86E+09	9.51E+09
BHR	Bahrain	3.45E+07	1.15E+07	6.25E+07	1.09E+08
BHS	Bahamas	7.80E+06	3.39E+07	1.67E+08	2.08E+08
BIH	Bosnia & Herzegovina	3.42E+08	4.16E+08	3.53E+09	4.29E+09
BLM	St. Barthélemy	1.75E+06	1.11E+06	2.23E+06	5.08E+06
BLR	Belarus	7.67E+08	1.23E+08	1.17E+10	1.26E+10

BLZ	Belize	8.33E+07	5.07E+07	6.40E+07	1.98E+08
BMU	Bermuda	3.85E+07	1.49E+06	1.00E+07	5.01E+07
BOL	Bolivia	2.75E+09	1.82E+08	4.80E+09	7.73E+09
BRA	Brazil	6.40E+10	7.47E+09	1.72E+11	2.44E+11
BRB	Barbados	1.89E+08	3.09E+06	1.22E+07	2.04E+08
BRN	Brunei	3.70E+07	9.37E+07	4.72E+07	1.78E+08
BTN	Bhutan	4.45E+08	1.02E+08	4.23E+07	5.89E+08
BWA	Botswana	1.14E+09	1.15E+07	3.29E+07	1.18E+09
CAF	Central African Republic	1.68E+09	4.46E+06	2.58E+07	1.71E+09
CAN	Canada	2.11E+09	9.10E+08	2.78E+10	3.08E+10
CCK	Cocos (Keeling) Islands	6.83E+04	1.40E+04	2.88E+03	8.52E+04
CHE	Switzerland	5.54E+08	4.88E+07	1.14E+10	1.21E+10
CHL	Chile	1.18E+09	5.16E+08	2.01E+10	2.18E+10
CHN	China	5.43E+11	1.43E+10	1.34E+12	1.89E+12
CIV	Côte d'Ivoire	8.14E+09	4.02E+08	1.70E+09	1.02E+10
CMR	Cameroon	1.51E+10	6.56E+08	2.24E+08	1.59E+10
COD	Congo - Kinshasa	3.07E+10	6.16E+08	6.34E+08	3.19E+10
COG	Congo - Brazzaville	1.98E+09	7.21E+07	5.89E+07	2.11E+09
COK	Cook Islands	4.28E+06	3.03E+06	2.64E+05	7.57E+06
COL	Colombia	3.02E+09	1.12E+09	3.87E+10	4.29E+10
COM	Comoros	7.50E+08	1.46E+07	5.44E+07	8.19E+08
CPV	Cape Verde	2.20E+07	1.00E+07	1.40E+08	1.72E+08
CRI	Costa Rica	2.97E+08	1.17E+09	1.29E+09	2.76E+09
CUB	Cuba	2.70E+09	5.42E+08	7.85E+09	1.11E+10
CUW	Curaçao	6.83E+05	3.70E+06	7.99E+07	8.43E+07
CXR	Christmas Island	4.57E+05	9.37E+04	7.16E+04	6.22E+05

CYM	Cayman Islands	9.03E+06	1.00E+07	1.89E+07	3.80E+07
CYP	Cyprus	2.10E+07	5.59E+07	5.19E+08	5.96E+08
CZE	Czechia	5.47E+08	3.65E+08	1.18E+10	1.27E+10
DEU	Germany	2.80E+09	9.04E+08	1.03E+11	1.06E+11
DJI	Djibouti	3.65E+08	1.27E+07	4.79E+07	4.26E+08
DMA	Dominica	3.45E+07	1.15E+07	1.96E+07	6.56E+07
DNK	Denmark	1.47E+08	8.51E+07	3.55E+08	5.88E+08
DOM	Dominican Republic	1.55E+09	7.76E+08	2.28E+09	4.60E+09
DZA	Algeria	2.05E+09	3.56E+08	3.54E+10	3.78E+10
ECU	Ecuador	1.16E+09	5.38E+08	6.62E+09	8.31E+09
EGY	Egypt	2.93E+09	1.80E+09	1.25E+11	1.30E+11
ERI	Eritrea	1.95E+09	4.78E+07	2.14E+08	2.21E+09
ESH	Western Sahara	5.70E+07	1.11E+07	2.35E+08	3.03E+08
ESP	Spain	1.46E+09	1.03E+08	5.27E+10	5.42E+10
EST	Estonia	1.97E+08	1.30E+07	1.76E+09	1.97E+09
ETH	Ethiopia	2.12E+10	3.86E+08	1.23E+09	2.28E+10
FIN	Finland	2.57E+09	1.33E+07	6.18E+09	8.76E+09
FJI	Fiji	7.79E+08	2.63E+07	9.31E+07	8.98E+08
FLK	Falkland Islands	6.29E+03	1.89E+04	3.18E+06	3.20E+06
FRA	France	2.16E+09	2.57E+09	6.62E+09	1.14E+10
FRO	Faroe Islands	4.57E+06	7.46E+06	2.96E+07	4.16E+07
FSM	Micronesia	1.65E+07	5.07E+06	1.46E+07	3.61E+07
GAB	Gabon	1.47E+09	3.07E+07	6.11E+08	2.11E+09
GBR	United Kingdom	1.61E+09	4.31E+08	6.61E+10	6.82E+10
GEO	Georgia	1.10E+09	8.92E+06	1.03E+09	2.14E+09
GGY	Guernsey	2.54E+06	2.70E+05	9.67E+07	9.95E+07

GHA	Ghana	1.22E+10	6.34E+08	1.27E+09	1.41E+10
GIB	Gibraltar	2.23E+06	1.51E+06	5.73E+07	6.10E+07
GIN	Guinea	7.38E+09	2.70E+08	3.34E+08	7.99E+09
GLP	Guadeloupe	6.71E+07	4.25E+07	9.93E+07	2.09E+08
GMB	Gambia	6.14E+08	3.07E+07	6.30E+07	7.08E+08
GNB	Guinea-Bissau	4.25E+08	1.89E+07	4.90E+07	4.92E+08
GNQ	Equatorial Guinea	1.41E+09	2.14E+07	1.44E+08	1.58E+09
GRC	Greece	4.09E+08	7.07E+08	1.30E+10	1.42E+10
GRD	Grenada	3.25E+07	1.11E+07	2.88E+07	7.24E+07
GRL	Greenland	2.03E+06	1.17E+06	2.49E+06	5.70E+06
GTM	Guatemala	6.04E+09	3.76E+08	9.25E+09	1.57E+10
GUF	French Guiana	6.95E+07	3.10E+07	5.80E+07	1.58E+08
GUM	Guam	9.42E+06	1.30E+07	2.92E+07	5.16E+07
GUY	Guyana	1.97E+08	1.29E+08	5.06E+07	3.77E+08
HKG	Hong Kong SAR China	1.27E+09	9.10E+07	1.29E+10	1.43E+10
HND	Honduras	2.84E+09	3.96E+08	3.11E+09	6.34E+09
HRV	Croatia	2.90E+08	3.31E+08	3.15E+09	3.77E+09
HTI	Haiti	6.23E+09	2.70E+08	9.59E+07	6.60E+09
HUN	Hungary	2.24E+09	2.95E+08	8.57E+09	1.11E+10
IDN	Indonesia	4.59E+10	4.07E+10	2.44E+10	1.11E+11
IMN	Isle of Man	1.43E+07	1.73E+06	1.26E+08	1.42E+08
IND	India	3.24E+11	6.92E+10	2.66E+11	6.59E+11
IRL	Ireland	6.47E+08	4.50E+08	4.32E+09	5.42E+09
IRN	Iran	1.52E+10	5.58E+07	6.65E+09	2.19E+10
IRQ	Iraq	4.81E+09	2.55E+09	1.05E+10	1.79E+10
ISL	Iceland	1.54E+07	7.20E+06	1.97E+07	4.23E+07

ISR	Israel	2.01E+08	2.67E+07	1.42E+10	1.44E+10
ITA	Italy	5.93E+09	4.11E+09	9.11E+10	1.01E+11
JAM	Jamaica	1.17E+09	1.50E+08	9.51E+08	2.27E+09
JEY	Jersey	5.19E+06	4.68E+05	1.48E+08	1.54E+08
JOR	Jordan	5.51E+06	1.17E+07	1.10E+08	1.27E+08
JPN	Japan	1.43E+10	7.83E+09	1.48E+10	3.69E+10
KAZ	Kazakhstan	2.54E+09	8.04E+07	3.54E+09	6.16E+09
KEN	Kenya	2.05E+10	3.06E+08	4.43E+09	2.52E+10
KHM	Cambodia	3.69E+09	1.22E+09	3.80E+09	8.72E+09
KIR	Kiribati	5.49E+07	5.37E+06	1.37E+07	7.40E+07
KNA	St. Kitts & Nevis	1.32E+07	2.83E+06	5.38E+06	2.15E+07
KOR	South Korea	3.18E+09	1.31E+08	2.77E+08	3.59E+09
KWT	Kuwait	1.05E+08	8.36E+07	4.28E+09	4.47E+09
LAO	Laos	7.41E+09	3.07E+08	1.07E+08	7.83E+09
LBN	Lebanon	1.07E+09	1.03E+07	4.24E+07	1.13E+09
LBR	Liberia	2.22E+09	9.81E+07	3.74E+07	2.36E+09
LBY	Libya	1.22E+09	8.10E+07	2.72E+08	1.58E+09
LCA	St. Lucia	2.53E+07	3.69E+07	2.03E+07	8.25E+07
LIE	Liechtenstein	2.88E+06	2.20E+05	1.89E+07	2.20E+07
LKA	Sri Lanka	1.45E+10	1.22E+08	1.26E+09	1.59E+10
LSO	Lesotho	1.64E+09	8.41E+06	6.65E+07	1.72E+09
LTU	Lithuania	3.89E+08	4.42E+07	5.01E+09	5.44E+09
LUX	Luxembourg	3.31E+07	1.42E+07	9.13E+08	9.60E+08
LVA	Latvia	2.28E+08	1.22E+07	2.41E+09	2.65E+09
MAC	Macao SAR China	4.45E+07	1.35E+06	6.48E+08	6.94E+08
MAF	Saint Martin (French part)	2.41E+06	1.24E+06	1.01E+07	1.37E+07

MAR	Morocco	6.47E+09	1.71E+09	3.16E+10	3.97E+10
MCO	Monaco	1.18E+06	5.36E+05	6.86E+07	7.03E+07
MDA	Moldova	2.06E+09	7.21E+07	1.89E+09	4.03E+09
MDG	Madagascar	1.62E+10	2.93E+08	2.76E+08	1.67E+10
MDV	Maldives	2.92E+07	2.49E+07	8.90E+07	1.43E+08
MEX	Mexico	1.18E+10	4.33E+09	1.20E+11	1.37E+11
MHL	Marshall Islands	1.31E+07	4.72E+06	1.10E+07	2.88E+07
MKD	North Macedonia	3.25E+08	4.95E+07	2.01E+09	2.39E+09
MLI	Mali	1.63E+10	1.69E+08	5.46E+08	1.70E+10
MLT	Malta	1.07E+07	1.04E+06	6.84E+08	6.96E+08
MMR	Myanmar (Burma)	5.93E+10	2.26E+09	7.43E+08	6.23E+10
MNE	Montenegro	5.34E+07	9.88E+07	3.69E+08	5.21E+08
MNG	Mongolia	2.22E+09	5.88E+06	6.47E+08	2.87E+09
MNP	Northern Mariana Islands	1.97E+06	3.79E+06	1.49E+07	2.06E+07
MOZ	Mozambique	9.45E+09	2.65E+08	4.61E+08	1.02E+10
MRT	Mauritania	2.91E+09	1.58E+08	1.88E+08	3.25E+09
MSR	Montserrat	1.63E+05	4.27E+04	2.08E+06	2.28E+06
MTQ	Martinique	1.90E+07	4.99E+07	1.13E+08	1.81E+08
MUS	Mauritius	6.48E+07	1.18E+06	6.93E+08	7.59E+08
MWI	Malawi	1.02E+10	7.39E+07	3.78E+08	1.07E+10
MYS	Malaysia	1.27E+09	1.99E+09	3.84E+09	7.10E+09
MYT	Mayotte	1.04E+08	1.55E+07	2.86E+07	1.48E+08
NAM	Namibia	4.34E+08	4.29E+06	4.43E+08	8.81E+08
NCL	New Caledonia	1.45E+08	1.17E+07	4.24E+07	1.99E+08
NER	Niger	1.61E+10	2.60E+08	4.63E+08	1.68E+10
NFK	Norfolk Island	3.75E+05	3.75E+05	3.75E+06	4.50E+06

NGA	Nigeria	6.86E+10	4.87E+09	1.86E+10	9.21E+10
NIC	Nicaragua	3.59E+09	1.76E+08	2.14E+09	5.91E+09
NIU	Niue	2.33E+04	2.28E+04	7.60E+05	8.06E+05
NLD	Netherlands	3.27E+08	3.77E+07	1.74E+10	1.78E+10
NOR	Norway	3.31E+08	2.34E+08	5.32E+08	1.10E+09
NPL	Nepal	1.37E+10	3.37E+09	3.54E+09	2.06E+10
NRU	Nauru	8.67E+06	1.06E+06	2.46E+06	1.22E+07
NZL	New Zealand	3.83E+08	1.63E+08	3.72E+08	9.18E+08
OMN	Oman	3.65E+08	9.21E+08	5.68E+08	1.85E+09
PAK	Pakistan	3.95E+10	5.95E+09	8.11E+10	1.27E+11
PAN	Panama	1.30E+09	3.28E+08	1.71E+09	3.34E+09
PCN	Pitcairn Islands	2.79E+04	1.88E+04	1.81E+03	4.85E+04
PER	Peru	8.30E+09	4.99E+08	2.30E+10	3.18E+10
PHL	Philippines	1.85E+10	1.56E+10	4.00E+09	3.80E+10
PLW	Palau	3.72E+05	2.18E+06	8.69E+06	1.12E+07
PNG	Papua New Guinea	6.23E+09	4.24E+08	1.53E+09	8.18E+09
POL	Poland	2.91E+09	2.08E+09	6.13E+09	1.11E+10
PRI	Puerto Rico	2.57E+08	4.00E+08	8.62E+08	1.52E+09
PRK	North Korea	1.29E+10	4.83E+08	1.34E+10	2.68E+10
PRT	Portugal	7.95E+08	5.98E+08	1.06E+10	1.20E+10
PRY	Paraguay	1.29E+09	2.49E+08	8.87E+08	2.43E+09
PSE	Palestinian Territories	1.64E+08	1.32E+08	2.99E+09	3.28E+09
PYF	French Polynesia	3.92E+07	4.45E+07	1.15E+08	1.99E+08
QAT	Qatar	5.03E+07	4.11E+07	2.00E+08	2.91E+08
REU	Réunion	5.09E+06	9.71E+06	1.41E+08	1.56E+08
ROU	Romania	1.58E+10	1.18E+08	3.43E+08	1.63E+10

RUS	Russia	1.90E+10	5.06E+08	7.37E+10	9.33E+10
RWA	Rwanda	5.06E+09	2.53E+07	4.17E+08	5.50E+09
SAU	Saudi Arabia	4.40E+08	1.36E+09	4.50E+09	6.30E+09
SDN	Sudan	2.24E+10	6.80E+08	8.75E+08	2.39E+10
SEN	Senegal	3.82E+09	5.80E+08	1.85E+09	6.25E+09
SGP	Singapore	2.30E+08	9.48E+08	8.72E+09	9.89E+09
SHN	St. Helena	1.37E+04	4.35E+04	7.52E+05	8.10E+05
SJM	Svalbard & Jan Mayen	5.23E+05	6.40E+04	3.37E+06	3.96E+06
SLB	Solomon Islands	3.98E+08	1.05E+07	2.62E+07	4.35E+08
SLE	Sierra Leone	6.18E+09	7.41E+07	5.87E+07	6.31E+09
SLV	El Salvador	2.86E+09	2.32E+08	3.05E+09	6.13E+09
SMR	San Marino	1.06E+06	1.09E+06	7.24E+06	9.39E+06
SOM	Somalia	5.75E+09	8.02E+07	1.35E+09	7.17E+09
SPM	St. Pierre & Miquelon	5.45E+04	1.42E+05	7.76E+06	7.95E+06
SRB	Serbia	4.73E+08	5.49E+08	5.25E+09	6.27E+09
SSD	South Sudan	8.20E+09	2.57E+08	1.27E+08	8.58E+09
STP	São Tomé & Príncipe	1.26E+08	9.00E+05	1.68E+07	1.44E+08
SUR	Suriname	8.53E+07	8.87E+07	1.07E+07	1.85E+08
SVK	Slovakia	4.35E+08	3.79E+08	4.09E+09	4.90E+09
SVN	Slovenia	4.72E+08	2.34E+08	4.81E+08	1.19E+09
SWE	Sweden	4.33E+08	3.70E+08	1.29E+10	1.37E+10
SWZ	Eswatini	9.11E+08	1.13E+07	1.54E+08	1.08E+09

SXM	Sint Maarten	1.42E+07	2.95E+06	9.98E+06	2.71E+07
SYC	Seychelles	1.26E+06	6.89E+06	3.69E+07	4.51E+07
SYR	Syria	2.16E+09	1.17E+08	1.34E+10	1.57E+10
TCA	Turks & Caicos Islands	3.33E+06	1.39E+06	1.28E+07	1.75E+07
TCD	Chad	7.72E+08	5.87E+06	2.43E+07	8.02E+08
TGO	Togo	2.45E+09	1.63E+08	7.13E+07	2.69E+09
THA	Thailand	2.83E+09	9.51E+09	5.35E+09	1.77E+10
TKL	Tokelau	2.05E+05	1.38E+05	6.49E+03	3.49E+05
TLS	Timor-Leste	7.06E+08	4.22E+07	1.20E+08	8.68E+08
TON	Tonga	6.30E+06	6.18E+06	1.20E+06	1.37E+07
TTO	Trinidad & Tobago	1.18E+08	2.19E+08	5.51E+08	8.88E+08
TUN	Tunisia	1.28E+09	3.49E+08	9.73E+09	1.14E+10
TUR	Turkey	5.42E+09	2.42E+09	9.99E+10	1.08E+11
TUV	Tuvalu	9.51E+05	1.24E+05	1.50E+06	2.58E+06
TWN	Taiwan	9.80E+09	2.57E+08	2.33E+10	3.33E+10
TZA	Tanzania	1.91E+10	4.56E+08	7.81E+08	2.03E+10
UGA	Uganda	1.18E+10	1.34E+08	6.16E+08	1.26E+10
UKR	Ukraine	2.48E+10	1.58E+08	3.02E+10	5.52E+10

URY	Uruguay	1.15E+08	1.64E+08	2.52E+09	2.80E+09
USA	United States	8.80E+09	1.49E+10	3.34E+11	3.58E+11
VAT	Vatican City	1.28E+06	4.51E+05	6.58E+07	6.75E+07
VCT	St. Vincent & Grenadines	3.69E+07	1.89E+07	4.12E+07	9.71E+07
VEN	Venezuela	1.68E+09	3.31E+08	1.01E+09	3.01E+09
VGB	British Virgin Islands	1.52E+06	3.91E+06	1.15E+07	1.69E+07
VIR	U.S. Virgin Islands	2.31E+06	8.66E+06	3.92E+07	5.01E+07
VNM	Vietnam	1.53E+10	1.67E+10	2.21E+09	3.42E+10
VUT	Vanuatu	1.53E+08	1.06E+07	2.92E+07	1.93E+08
WLF	Wallis & Futuna	2.37E+05	1.60E+05	1.76E+05	5.72E+05
WSM	Samoa	1.04E+07	1.55E+07	2.29E+06	2.82E+07
YEM	Yemen	3.03E+09	5.66E+08	9.77E+09	1.34E+10
ZAF	South Africa	1.48E+10	2.19E+08	4.25E+10	5.75E+10
ZMB	Zambia	6.48E+09	1.38E+08	2.24E+09	8.86E+09
ZWE	Zimbabwe	4.85E+09	1.24E+08	5.65E+09	1.06E+10

S5 Table Values calculated and used in Fig. 2 of the main text for wastewater N input levels into EEZs around the world.

[1] Mayorga E, Seitzinger S P, Harrison J A, Dumont E, Beusen A H W, Bouwman A F, Fekete B M, Kroeze C and Van Drecht G 2010 Global Nutrient Export from WaterSheds 2 (NEWS 2): Model development and implementation *Environmental Modelling & Software* **25** 837–53
