# Supplementary material for: Mapping global inputs and impacts from of human sewage in coastal ecosystems
Source: PLoS One. 2021 Nov 10;16(11):e0258898. doi: 10.1371/journal.pone.0258898 (PMC8580218; doi:10.1371/journal.pone.0258898)
Supplement: Attachment — Submitted filename: Review of PONE-D-21-15046.docx [file pone.0258898.s002.docx]

Review of PONE-D-21-15046

Mapping global inputs of and impacts from human sewage in coastal ecosystems

General

1. This is an important contribution, and my comments are mostly concerned with grammar.

2. Define “pourpoint” upon first use (i.e., the point at which water flows from the watershed to the coastal ocean).

3. Replace “environmental health” with “ecological health” throughout.

Specific

1. Please reference the following along with reference #2:

Malone, T.C. and A. Newton. 2020. The globalization of cultural eutrophication in the coastal ocean: Causes and consequences. Front. Mar. Sci. 7:670. doi: 10.3389/fmars.2020.00670

2. p.3, line 1: This should read “…risk from wastewater **has** largely **occurred** in isolation [26].”

3. p.3, line 9: This should read “…focus primarily on the largest watersheds…”

4. p. 3, line 15: “Primarily” is repeated twice. Delete one.

5. p. 3, line 20: This should read “…can provide context for specific details, such as how fecal…”

6. p. 3, line 22: This should read “…[39], e.g., global pathogen wastewater…”

7. p. 3, line 24: What are “sub-national policy recommendations”?

8. p. 11, line 17: “…alone with **climate change** and **other** anthropogenic stressors [1].” Replace “climate change” with “climate driven stressors (e.g., ocean warming, acidification and sea level rise).

9. p. 13, line 3: This should read “…ecosystems to take up N but do…”

10. p. 14, line 3: Replace “known” with “observed”.

11. p. 15, line 4: Delete “vast majority of the”.
